# Improvement of the Chondrocyte-Specific Phenotype upon Equine Bone Marrow Mesenchymal Stem Cell Differentiation: Influence of Culture Time, Transforming Growth Factors and Type I Collagen siRNAs on the Differentiation Index

**DOI:** 10.3390/ijms19020435

**Published:** 2018-02-01

**Authors:** Thomas Branly, Romain Contentin, Mélanie Desancé, Thibaud Jacquel, Lélia Bertoni, Sandrine Jacquet, Frédéric Mallein-Gerin, Jean-Marie Denoix, Fabrice Audigié, Magali Demoor, Philippe Galéra

**Affiliations:** 1 Normandie Univ, UNICAEN, BIOTARGEN, 14000 Caen, France; tbranly@gmail.com (T.B.); romaincontentin@hotmail.fr (R.C.); melanie359@hotmail.fr (M.D.); jacquel.thibaud@gmail.com (T.J.); magali.demoor@unicaen.fr (M.D.); 2 Center of Imaging and Research on Locomotor Affections in Equines, Ecole Vétérinaire d’Alfort, Université Paris-Est, 14430 Goustranville, France; lelia.bertoni@vet-alfort.fr (L.B.); sandrine.jacquet@vet-alfort.fr (S.J.); jmdenoix@vet-alfort.fr (J.-M.D.); fabrice.audigie@vet-alfort.fr (F.A.); 3 Institute for Biology and Chemistry of Proteins, CNRS, UMR 5305 Laboratory of Tissue Biology and Therapeutic Engineering, Université Claude Bernard-Lyon 1, Université de Lyon, 69367 Lyon CEDEX 07, France; f.mallein-gerin@ibcp.fr

**Keywords:** horse, mesenchymal stem cells, chondrocytes, bone marrow, cartilage engineering, *Col1a1-Col1a2-HtrA1* siRNAs, osteoarthritis, chondral defects, chondrogenesis, extracellular matrix

## Abstract

Articular cartilage is a tissue characterized by its poor intrinsic capacity for self-repair. This tissue is frequently altered upon trauma or in osteoarthritis (OA), a degenerative disease that is currently incurable. Similar musculoskeletal disorders also affect horses and OA incurs considerable economic loss for the equine sector. In the view to develop new therapies for humans and horses, significant progress in tissue engineering has led to the emergence of new generations of cartilage therapy. Matrix-associated autologous chondrocyte implantation is an advanced 3D cell-based therapy that holds promise for cartilage repair. This study aims to improve the autologous chondrocyte implantation technique by using equine mesenchymal stem cells (MSCs) from bone marrow differentiated into chondrocytes that can be implanted in the chondral lesion. The optimized protocol relies on culture under hypoxia within type I/III collagen sponges. Here, we explored three parameters that influence MSC differentiation: culture times, growth factors and RNA interference strategies. Our results suggest first that an increase in culture time from 14 to 28 or 42 days lead to a sharp increase in the expression of chondrocyte markers, notably type II collagen (especially the IIB isoform), along with a concomitant decrease in HtrA1 expression. Nevertheless, the expression of type I collagen also increased with longer culture times. Second, regarding the growth factor cocktail, TGF-β3 alone showed promising result but the previously tested association of BMP-2 and TGF-β1 better limits the expression of type I collagen. Third, RNA interference targeting *Col1a2* as well as *Col1a1* mRNA led to a more significant knockdown, compared with a conventional strategy targeting *Col1a1* alone. This chondrogenic differentiation strategy showed a strong increase in the *Col2a1*:*Col1a1* mRNA ratio in the chondrocytes derived from equine bone marrow MSCs, this ratio being considered as an index of the functionality of cartilage. These data provide evidence of a more stable chondrocyte phenotype when combining *Col1a1* and *Col1a2* siRNAs associated to a longer culture time in the presence of BMP-2 and TGF-β1, opening new opportunities for preclinical trials in the horse. In addition, because the horse is an excellent model for human articular cartilage disorders, the equine therapeutic approach developed here can also serve as a preclinical step for human medicine.

## 1. Introduction

Healthy hyaline cartilage is composed in particular of type II, IX and XI collagens and aggrecans [1]. Type II collagen is the most quantitatively abundant collagen type in the cartilaginous extracellular matrix (ECM) and alternative splicing generates different isoforms. The longest type IIA isoform is expressed in early chondrogenesis and in mesenchymal stem cells (MSCs) undergoing chondrogenic differentiation, while the IIB isoform, devoid of amino acids encoded by exon II, is expressed in mature chondrocytes, the main cell type of healthy hyaline articular cartilage [2,3]. The switch from type IIA collagen to type IIB during chondrogenesis is a sign of differentiation into mature chondrocytes [4]. Furthermore, a transmembrane proteoglycan, Snorc (Small NOvel Rich in Cartilage), has been described as a marker of the chondrocyte phenotype [5]. Snorc is expressed during embryonic mouse limb chondrogenesis and appears to be a cartilage specific marker because it has not been detected in several mouse tissues such as brain, heart, liver or skin. Additionally, Snorc colocalizes with BMP-2 and type IIA procollagen in the growth plate of mice, suggesting a potential effect of BMP-2 on Snorc expression. BMP-2 increases Snorc mRNA expression in the same pattern of those of type II collagen, aggrecan and Sox9 in a mouse limb bud micromass culture model of chondrogenesis [5].

Osteoarthritis (OA) is the most common degenerative bone and joint disease in humans worldwide. It is characterized by the degradation of articular cartilage, leading to exposure of the bone and a loss of motor capacity due to the onset of pain. Chondrocytes synthesize a fibrocartilage composed mainly of type I collagen in OA. This fibrocartilage is rapidly degraded [6] and then cells enter apoptosis [7]. Type I collagen is an heterotrimer composed of two α1(I) chains and one α2(I) chain, encoded respectively by the *COL1A1* and *COL1A2* genes (for review, [8]). This collagen isotype is expressed in many tissues such as skin, tendon or bone but not in healthy hyaline articular cartilage. Moreover, the serine protease HtrA1, which is overexpressed in OA, causes proteolysis of aggrecan and the decrease in the accumulation of glycosaminoglycans [9]. In addition, HtrA1 appears to induce the degradation of the receptors of the TGF-β family members, which in turn cannot trigger chondrogenic signals necessary for the differentiation of chondrocytes and the maintenance of the chondrocyte phenotype [10] (for review [11]).

Due to its status as a domesticated animal and as a high-level athlete, the horse is an excellent model for studying human locomotor disorders. Both species share very close similarities such as the structure, biochemistry, thickness and cellularity of cartilage [12,13]. The incentives for developing articular cartilage therapies in the horse are especially high because locomotor disorders are the leading cause in the decline of the performance of sport horses, occurring in 80% of cases according to a New Zealand study [14]. However, therapeutic strategies for OA are currently very limited and are mainly limited to symptomatic treatments. Given these problems, new therapeutic strategies in human medicine have emerged, including autologous chondrocyte transplantation (ACT), consisting of the removal of healthy articular chondrocytes from a non-bearing area of the joint, for in vitro amplification and re-implantation under a periosteum membrane [15]. However, this methodological approach has many limitations, particularly due to the synthesis of fibrocartilage by the implanted chondrocytes and the tendency of the neoconstruct to detach [16].

Subsequent generations of ACT seek to improve this method with, in particular, the use of mesenchymal stem cells (MSCs) to obtain a sufficient number of cells for implantation [17]. MSCs have the ability to differentiate into chondrocytes [18] and do not pose any specific risks, especially because they do not cause the formation of teratocarcinomas in vivo, unlike embryonic stem cells [19]. The use of a biomaterial as a 3D culture model provides a directly implantable cartilaginous substitute. The choice of the biomaterial proves to be one of the critical factors in ACT strategies, because they influence cell behaviour and the quality of the ECM newly synthesized by the cells [20] (for review, [11]). The growth factors used to induce chondrogenic differentiation of MSCs also represent a major parameter in the implementation of such strategies. Thus, treatment of MSCs with a cocktail consisting of bone morphogenetic protein (BMP)-2 and transforming growth factor-β1 (TGF-β1) promotes MSC differentiation into chondrocytes [21]. However, the in vitro use of TGF-β (isoforms TGF-β1 or TGF-β3) induces the expression of type X collagen, a hypertrophy marker which is not found in mature articular chondrocytes [22]. This constraint can be overcome, however, by the culture of MSCs in hypoxia. Low oxygen tension, mimicking the physioxia of the chondrocytes, decreases the expression of *Col10a1* in MSCs from bone marrow (BM) undergoing chondrogenic differentiation [23]. A human chondrogenic MSC differentiation protocol was developed in the laboratory and includes all of these mediators of differentiation [24,25,26]. This protocol was subsequently transferred to the equine model and we showed that a culture of equine BM-MSCs cultured in type I/III collagen sponges in the presence of BMP-2 and TGF-β1 for 14 days leads to the production of a hyaline-type neocartilaginous substitute [27]. Furthermore, an RNA interference strategy using siRNA targeting *Col1a1* and *Htra1* causes significant inhibition of the expression of their target transcripts and corresponding proteins. We also showed that the use of BMP-2 and TGF-β1, even though it leads to an increase in the steady-state amounts of *Htra1* mRNAs, ultimately decreases the expression of the HtrA1 protein [27].

The aim of the present study was to explore three parameters that can help improve this culture procedure and thereby increase the functional index of chondrocytes derived from equine BM-SCs. First, to do so, we compared the effects of TGF-β3 and TGF-β3 + BMP-2 with those of the BMP-2 + TGF-β1 cocktail. Second, we studied the effect of the extension of culture time from 14 to 42 days because some studies have suggested that a longer culture period may stabilize the chondrocyte phenotype by increasing the overall amount of ECM proteins and their maturation [28,29]. Third, we tested new siRNAs targeting *Col1a1* but also *Col1a2* mRNAs to optimize the inhibition of protein synthesis of type I collagen in our neocartilage substitute. Our rationale was that type I collagen is made up of two α1(I) and one α2(I) chains respectively encoded by the *COL1A1* and *COL1A2* genes which are co-ordinately regulated at the transcriptional level under physiopathological conditions. In most circumstances, the two protein chains of type I collagen are synthesized in a 2:1 stoichiometry and the same stoichiometry is also observed for the steady-state levels of the corresponding mRNAs in human, mouse and chick fibroblasts and for their rates of synthesis [30,31,32,33].

## 2. Result

### 2.1. Kinetic Study of the BMP-2 and TGF-β1 Effect on the Phenotypic Status of Chondrocytes Derived from BM-MSCs

**Figure 1:**
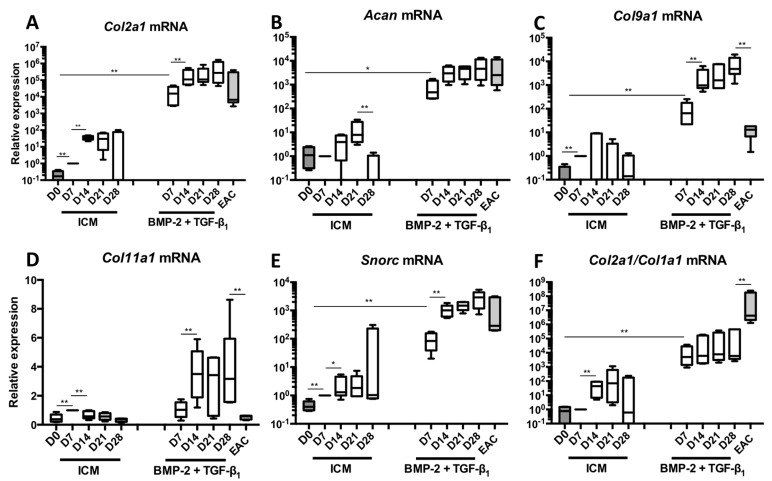
Kinetic study of mRNA expression encoding the markers specific to articular cartilage during chondrogenic differentiation of MSCs. Equine MSCs derived from bone marrow were amplified and seeded in collagen sponges at P4 (*n* = 5). They were then grown in hypoxia (3% O_2_) for 7, 14, 21 and 28 days (D7, D14, D21 and D28, respectively) in the presence of incomplete chondrogenic medium (ICM) alone or enriched with BMP-2 (50 ng/mL) and TGF-β1 (10 ng/mL) (BMP-2 + TGF-β1). The D0 condition corresponds to stem cells cultured in monolayer at P4 with the amplification medium and the equine articular chondrocyte (EAC) condition corresponds to the mRNAs extracted from EACs obtained after enzymatic digestion of healthy cartilage. mRNAs encoding *Col2a1* (**A**); *Acan* (**B**); *Col9a1* (**C**); *Col11a1* (**D**) and *Snorc* (**E**) were estimated by RT-qPCR after normalization relative to the *β-actin* reference gene. The *Col2a1*:*Col1a1* ratio (**F**) is shown. The results are represented as box plots (median, quartiles, extreme values) and the significance of the values was tested using the Mann-Whitney test (* *p* < 0.05, ** *p* < 0.01).

**Figure 2:**
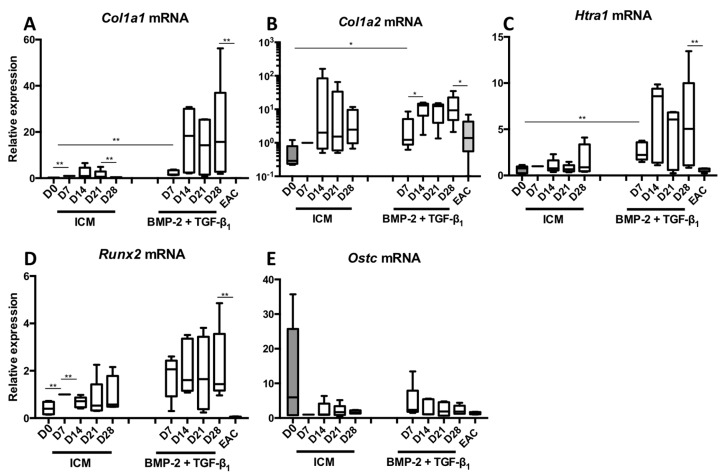
Kinetic study of mRNA expression encoding nonspecific markers of hyaline articular cartilage during chondrogenic differentiation of MSCs. Equine MSCs derived from bone marrow were amplified and seeded in collagen sponges at P4 (*n* = 5). They were then grown in hypoxia (3% O_2_) for 7, 14, 21 and 28 days (D7, D14, D21 and D28, respectively) in the presence of incomplete chondrogenic medium (ICM) alone or enriched with BMP-2 (50 ng/mL) and TGF-β1 (10 ng/mL) (BMP-2 + TGF-β1). The D0 condition corresponds to stem cells cultured in monolayer at P4 with the amplification medium and the equine articular chondrocyte (EAC) condition corresponds to the mRNAs extracted from EACs obtained after enzymatic digestion of healthy cartilage. mRNA levels of *Col1a1* (**A**), *Col1a2* (**B**), *Htra1* (**C**), *Runx2* (**D**) and *Ostc* (**E**) were estimated by RT-qPCR after normalization relative to the *β-actin* reference gene. The results are represented as box plots (median, quartiles, extreme values) and the significance of the values was tested using the Mann-Whitney test (* *p* < 0.05, ** *p* < 0.01).

BM-MSCs were cultured in hypoxia in type I/III collagen sponges in a control medium (ICM) or in the presence of a BMP-2 + TGF-β1 cocktail. MSCs were cultured for 7, 14, 21 and 28 days to evaluate the steady-state amounts of mRNAs that encode markers specific to the chondrocyte phenotype (Figure 1). The nonspecific markers of chondrocytes, as well as the markers of hypertrophy or of the terminal differentiation of endochondral ossification were also evaluated (Figure 2). Our data show that, already at 7 days and compared with the control, the use of BMP-2 + TGF-β1 significantly increased the mRNA levels of *Col2a1* (Figure 1A), *Acan* (Figure 1B), *Col9a1* (Figure 1C), *Col11a1* (Figure 1D), *Snorc* (Figure 1E) and the *Col2a1*:*Col1a1* ratio (Figure 1F). Furthermore, between 14 and 28 days of culture, there was a relatively high increase in the mRNA levels of these specific markers, compared with the 7-day culture, except for *Col11a1* messengers which stabilized after 14 days of culture. These results demonstrate that culturing cells for 28 days leads to better chondrogenic differentiation of BM-MSCs, with *Col2a1*, *Acan* and *Snorc* mRNA amounts comparable to those found in equine articular cartilage (EAC). The amounts of *Col9a1* and *Col11a1* mRNAs were also found in greater quantities than those found in EAC. However, the *Col2a1*:*Col1a1* ratio remained stable between 7 and 28 days of culture. This is attributed to the fact that *Col1a1* mRNAs levels increased at 14 days of culture and maintained up to 28 days (Figure 2A). The amounts of *Col1a1* transcripts were strongly enhanced after 14 days of culture compared with 7 days. Thereafter, the *Col1a1* mRNAs were stable between 14 and 28 days. A similar pattern was observed for *Col1a2* (Figure 2B) as well as *Htra1* (Figure 2C).

Regarding *Col1a2* mRNAs, the treatment of BM-MSCs with the chondrogenic factors did not seem to substantially induce its expression compared with the *Col1a1* and *Htra1* mRNAs compared with the ICM control. Regarding *Runx2*, an early marker of chondrocyte hypertrophy, its expression, initially induced in control conditions and even more when the cells were cultured in the presence of BMP-2 + TGF-β1, tended to decrease after 14 days of culture. Its expression remains stable between D14 and D28. However, *Runx2* expression still remained higher than that found in EACs (Figure 2D). The mRNA steady-state amounts of *Ostc* did not seem modulated in these culture conditions and remained at the same order of magnitude as what is found in EACs (Figure 2E).

**Figure 3:**
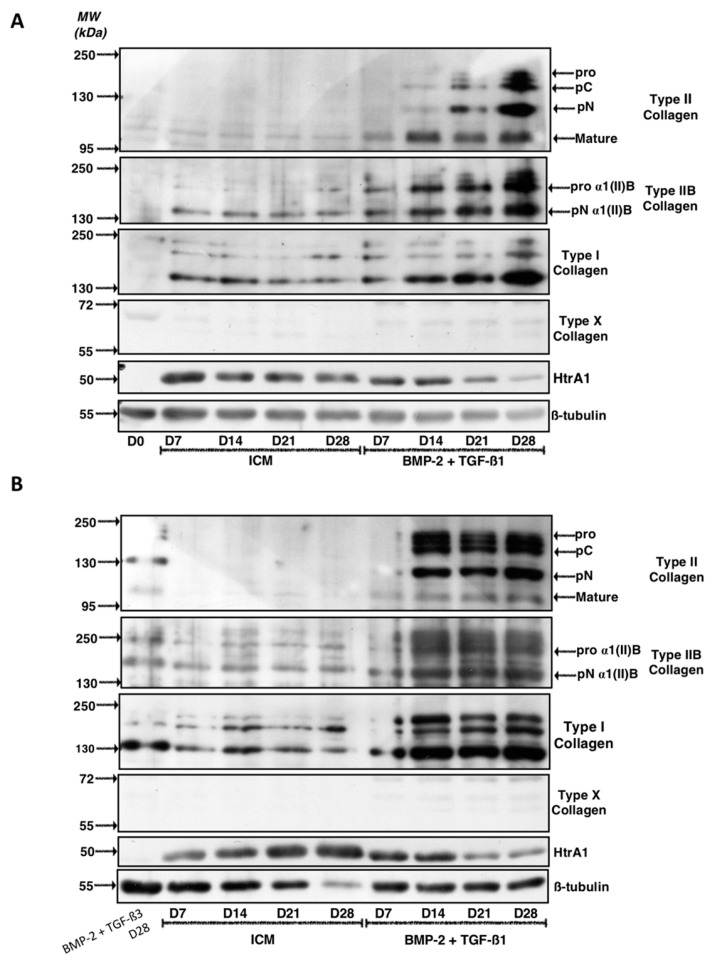
Kinetic study of the expression of cartilage matrix proteins during chondrogenic differentiation of MSCs. Equine MSCs derived from bone marrow were amplified and seeded in collagen sponges at P4 (*n* = 3). They were then grown in hypoxia (3% O_2_) for 7, 14, 21 and 28 days (D7, D14, D21 and D28, respectively) in the presence of incomplete chondrogenic medium (ICM) alone or enriched with BMP-2 (50 ng/mL) and TGF-β1 (10 ng/mL) (BMP-2 + TGF-β1). The sponges were harvested and crushed. The total protein extracts were separated by electrophoresis on a 10% acrylamide gel under denaturing conditions. The proteins were then transferred to a PVDF membrane which was incubated with the anti-collagen II, anti-collagen IIB, anti-collagen I, anti-collagen X, anti-HtrA1 or anti-β-tubulin primary antibody. The molecular weight marker (MW kDa) is indicated on the left. Two representative experiments from two strains of MSCs are shown in panels (**A**,**B**).

Western blots were performed to confirm that the results obtained at the mRNA level are correlated with the amounts of the corresponding ECM proteins in our biological substitute (Figure 3 and Figure S1 for the quantitative of the membrane signals). Several strains were evaluated and two types of results were systematically observed as described for the two representative BM-MSCs strains presented. For the first strain (Figure 3A), the specific bands of type II collagen (immature and mature forms), absent in the ICM condition, increased in intensity for culture times between 7 and 28 days, when BMP-2 + TGF-β1 were added. Culturing for 28 days led to the expression of the highest amounts of all the maturation forms of type II collagen. Strictly identical patterns were observed for type IIB collagen, the specific phenotypic marker of mature chondrocytes. Nevertheless, the addition of BMP-2 + TGF-β1 also strongly induced type I collagen protein synthesis and the effect increased with culture time. At 28 days, the newly synthesized ECM appeared to contain a maximum of type I collagen, particularly the low-molecular-weight mature form.

Regarding HtrA1, culture of BM-MSCs in ICM induces its protein synthesis compared to MSCs at day 0 (D0) and the increase in culture time did not seem to modulate its expression in the neocartilage. In contrast, the treatment of the cells with BMP-2 + TGF-β1 decreased HtrA1 expression compared with ICM at each respective culture time and its expression was almost blocked at 28 days.

Type X collagen, one of the major phenotypic markers of chondrocyte hypertrophy, was not detected in our culture conditions in ICM nor in BMP-2 + TGF-β1 experimental conditions. This suggests that the chondrogenic factors do not induce hypertrophy of the chondrocytes derived from BM-MSCs.

The results obtained with the second BM-MSC strain studied differed slightly, although the final effects of the different growing conditions were similar (Figure 3B). Thus, in these culture conditions, although the addition of BMP-2 + TGF-β1 led to the synthesis of type II collagen and more particularly of type IIB collagen, the protein levels were already maximal at 14 days and did not change significantly between 14 and 28 days of cell culture. Similarly, the protein levels of type I collagen induced by the addition of growth factors compared with ICM were maximal at 14 days and stabilized between 14 and 28 days of culture.

Regarding HtrA1, however, the same trends were observed in both strains and the cells cultured in the presence of BMP-2 + TGF-β1 for 21 and 28 days showed traces of this serine protease compared with all other experimental conditions studied. Nevertheless, in this strain, HtrA1 expression increased with culture time in the ICM control conditions, unlike the first strain.

Similar to the first strain of BM-MSCs, type X collagen was not expressed in any of the experimental conditions studied here.

### 2.2. TGF-β3 Effect on the Differentiation Status of the Chondrocytes Derived from BM-MSCs

**Figure 4:**
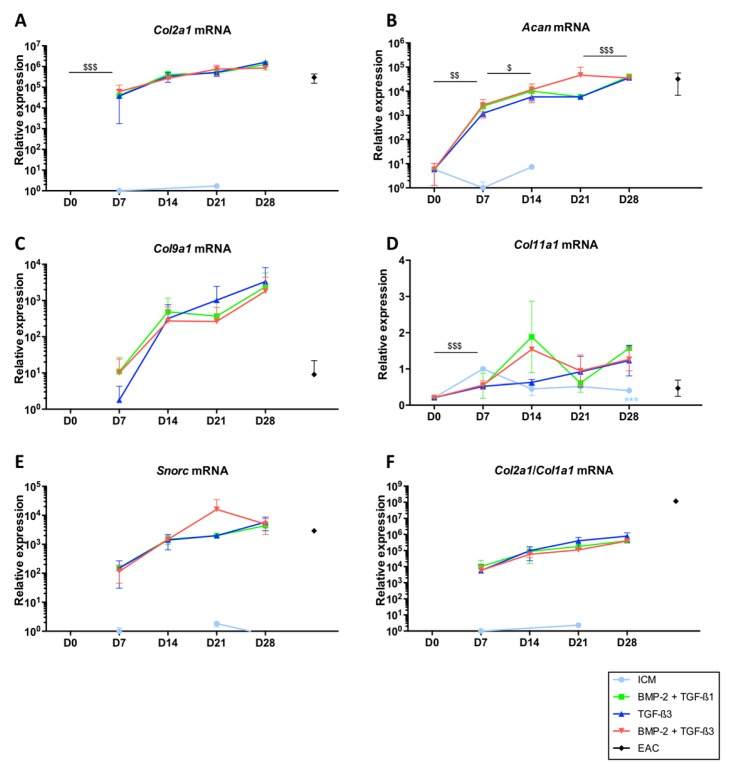
Expression of mRNAs of markers specific to articular cartilage during chondrogenic differentiation of MSCs cultured in the presence of BMP-2 + TGF-β1 or TGF-β3 alone or in combination with BMP-2. Equine MSCs derived from bone marrow were amplified and seeded in collagen sponges at P4 (*n* = 3). They were then grown in hypoxia (3% O_2_) for 7, 14, 21 and 28 days (D7, D14, D21 and D28, respectively) in the presence of incomplete chondrogenic medium (ICM) alone or enriched with BMP-2 (50 ng/mL) and TGF-β1 (10 ng/mL) (BMP-2 + TGF-β1), TGF-β3 (10 ng/mL) or BMP-2 (50 ng/mL) and TGF-β3 (10 ng/mL) (BMP-2 + TGF-β3). The D0 condition corresponds to stem cells cultured in monolayer at P4 with the amplification medium and the equine articular chondrocyte (EAC) condition corresponds to the mRNAs extracted from EACs obtained after enzymatic digestion of healthy cartilage. mRNA amounts of *Col2a1* (**A**); *Acan* (**B**); *Col9a1* (**C**) *Col11a1* (**D**) and *Snorc* (**E**) were estimated by RT-qPCR after normalization relative to the *β-actin* reference gene. The *Col2a1*:*Col1a1* ratio is shown (**F**). A representative experiment is shown. The results present mean values ± SD for the different experimental conditions studied and the significance of the values was tested using Student’s *t*-test comparing different groups: firstly, a given culture condition (ICM, TGF-β3, BMP-2 + TGF-β3) compared with BMP-2 + TGF-β1, at the same time of culture (*** *p* < 0.001); secondly the TGF-β3 condition at a given culture time, compared to the previous culture time ($ *p* < 0.05, $$ *p* < 0.01, $$$ *p* < 0.001).

**Figure 5:**
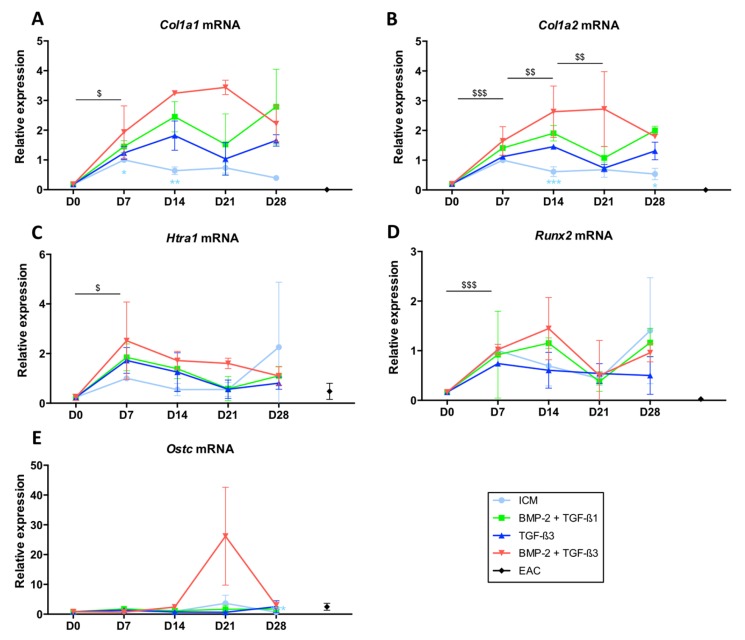
Expression of mRNAs encoding markers nonspecific to hyaline articular cartilage when MSCs were cultured in the presence of BMP-2 and TGF-β1 or TGF-β3 used alone or in combination with BMP-2. Equine MSCs derived from bone marrow were amplified and seeded in collagen sponges at P4 (*n* = 3). They were then grown in hypoxia (3% O_2_) for 7, 14, 21 and 28 days (D7, D14, D21 and D28, respectively) in the presence of incomplete chondrogenic medium (ICM) alone or supplemented with BMP-2 (50 ng/mL) and TGF-β1 (10 ng/mL) (BMP-2 + TGF-β1), TGF-β3 (10 ng/mL) or BMP-2 (50 ng/mL) and TGF-β3 (10 ng/mL) (BMP-2 + TGF-β3). The D0 condition corresponds to stem cells cultured in monolayer at P4 with the amplification medium and the equine articular chondrocyte (EAC) condition corresponds to the mRNAs extracted from EACs obtained after enzymatic digestion of healthy cartilage. mRNA levels of *Col1a1* (**A**); *Col1a2* (**B**), *Htra1* (**C**), *Runx2* (**D**) and *Ostc* (**E**) were estimated by RT-qPCR after normalization relative to the *β-actin* reference gene. The results present mean values ± SD for the different experimental conditions studied and the significance of the values was tested using Student’s *t*-test comparing different groups: firstly, a given culture condition (ICM, TGF-β3, BMP-2 + TGF-β3) compared with BMP-2 + TGF-β1, at the same time of culture (* *p* < 0.05, ** *p* < 0.01, *** *p* < 0.001); secondly the TGF-β3 condition at a given culture time, compared to the previous culture time ($ *p* < 0.05, $$ *p* < 0.01, $$$ *p* < 0.001).

**Figure 6:**
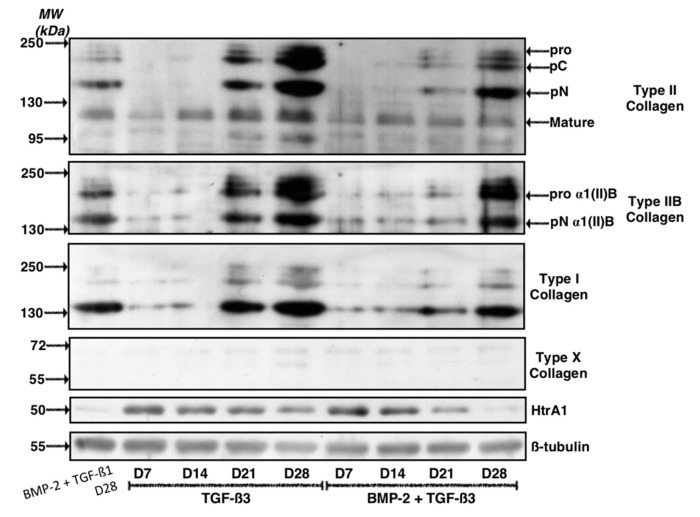
Kinetic study of the expression of cartilage matrix proteins after culture in the presence of BMP-2 and TGF-β3. Equine MSCs derived from bone marrow were amplified and seeded in collagen sponges at P4 (*n* = 4). They were then cultured in hypoxia (3% O_2_) for 7, 14, 21 and 28 days (D7, D14, D21 and D28, respectively) in the presence of incomplete chondrogenic medium (ICM) supplemented with TGF-β3 (10 ng/mL) alone or in combination with BMP-2 (50 ng/mL) (BMP-2 + TGF-β3). The sponges were harvested and crushed. The total protein extracts were separated by electrophoresis on a 10% acrylamide gel under denaturing conditions. The proteins were then transferred to a PVDF membrane which was incubated with the anti-collagen II, anti-collagen IIB, anti-collagen I, anti-collagen X, anti-HtrA1 or anti-β-tubulin primary antibody. The molecular weight (MW kDa) is indicated on the left. A representative experiment from different strains of MSCs is shown.

**Figure 7:**
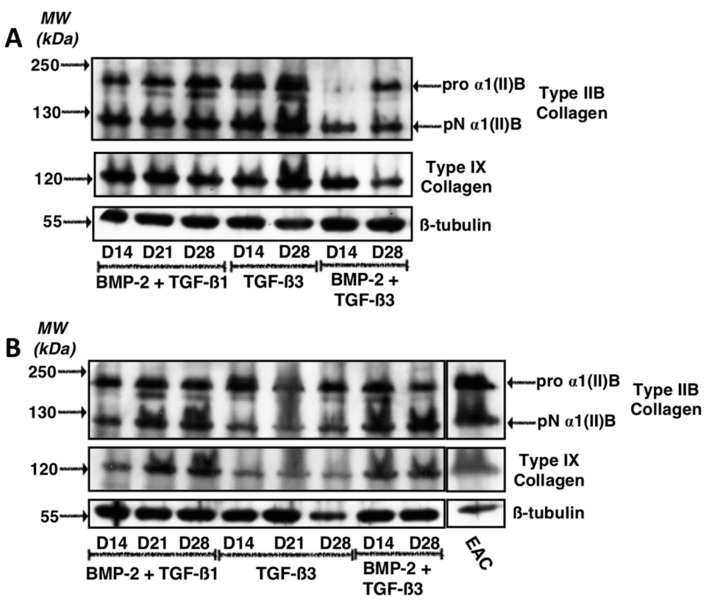
Expression of type IX and IIB collagens after culture in the presence of BMP-2 and TGF-β1 or TGF-β3 used alone or in combination with BMP-2. Equine MSCs derived from bone marrow were amplified and seeded in collagen sponges at P4 (*n* = 2). They were then cultured in hypoxia (3% O_2_) for 14, 21 and 28 days (D14, D21 and D28, respectively) in the presence of incomplete chondrogenic medium (ICM) supplemented with TGF-β3 (10 ng/mL) alone or in combination with BMP-2 (50 ng/mL) (BMP-2 + TGF-β3) or BMP-2 (50 ng/mL) and TGF-β1 (10 ng/mL) (BMP-2 + TGF-β1). The sponges were harvested and crushed. The total protein extracts were separated by electrophoresis on 10% acrylamide gel under denaturing conditions. The proteins were then transferred to a membrane which was incubated with the anti-collagen IIB, anti-collagen IX or anti-β-tubulin primary antibody. The molecular weight (MW kDa) is indicated on the left. Two representative experiments from two strains of MSCs are shown in panels (**A**,**B**). EAC: equine articular cartilage.

To assess the influence of TGF-β3 on the differentiation of BM-MSCs into chondrocytes, cells were grown in hypoxia for 7, 14, 21 and 28 days in the conditions described above, namely in ICM (control), in the presence of BMP-2 and TGF-β1 (BMP-2 + TGF-β1 cocktail) and in the presence of only TGF-β3 or in combination with BMP-2 (BMP-2 + TGF-β3 cocktail). The same phenotypic markers described above were evaluated by RT-qPCR (Figure 4 and Figure 5) and by western blot (Figure 6 and Figure 7). Thus, our results confirmed the trends previously established, namely that the use of BMP-2 + TGF-β1 induced the synthesis of *Col2a1* (Figure 4A), *Acan* (Figure 4B), *Col9a1* (Figure 4C), *Snorc* (Figure 4E) and significantly increased the *Col2a1*:*Col1a1* ratio (Figure 4F) in a time-dependent manner. In ICM, the mRNAs encoding these markers were not expressed by the cells, or only very weakly. It should be noted that when no data is present on the logarithmic representation on graphics for ICM and D0 conditions, it means that the gene was not detected during the experiment (quantity equal to 0).

Regarding *Col11a1* (Figure 4D), the addition of growth factors modestly increased its mRNA amounts, to a lesser extent than the effect observed for mRNA encoding the other markers specific to the chondrocyte phenotype. No significant differences were observed when the cells were cultured in the presence of BMP-2 + TGF-β1, TGF-β3 alone or BMP-2 + TGF-β3.

Regarding the *Col1a1* and *Col1a2* mRNAs (Figure 5A,B, respectively), the patterns were identical in the different growing conditions. Thus, culture in ICM led to the synthesis of smaller amounts of their respective messengers compared with culture conditions in the presence of growth factors and appeared to decrease with the duration of culture time. However, ICM did not lead to the protein synthesis of type II collagen and therefore is not favourable for the synthesis of a hyaline-like neocartilage substitute.

The addition of TGF-β3 showed the lowest mRNA synthesis of *Col1a1* and *Col1a2*, without a statistically significant effect. Conversely, the maximal expression of the *Col1a1* and *Col1a2* mRNAs was observed for the BMP-2 + TGF-β3 cocktail at 14 and 21 days of culture but culturing them for 28 days decreased their expression, becoming comparable to those found in the other combinations of growth factors studied.

The expression of *Htra1* was not modulated according to the growth factors used during the culture and its levels of mRNAs tended to decrease with culture time, becoming minimal after 28 days of culture (Figure 5C). In addition, *Runx2* was not induced by the addition of growth factors and its expression did not vary with culture time (Figure 5D), as was case for the expression of *Ostc* (Figure 5E).

The western blot analyses, however, revealed differences that were not highlighted by the RT-qPCR experiments. Our results indicate that the use of TGF-β3, alone or in combination with BMP-2 led to the protein synthesis of type II collagen and the effect was even greater with increasing culture time (Figure 6). The addition of TGF-β3 alone, however, showed higher synthesis of type II collagen compared with the BMP-2 + TGF-β3 or the BMP-2 + TGF-β1 cocktail at 28 days. The trend was similar when considering the type IIB collagen. As described above, type X collagen was not detected in our experimental conditions. Regarding HtrA1, its synthesis decreased with culture time and protein expression was at its lowest in the BMP-2 + TGF-β3 cocktail after 28 days of culture. Its protein levels were comparable to those found in the BMP-2 + TGF-β1 culture conditions after D28. Finally, in regard to the protein levels of type I collagen, the band intensities again suggest an increase in protein levels as a function of culture time with a maximum expression after 28 days of culture. However, its expression was highest when the cells were cultured in the presence of TGF-β3 alone, for mature and immature forms of this collagen. Type I collagen expression was more restricted when the cells were cultured in the presence of BMP-2 for 28 days, in association with TGF-β1 or of TGF-β3. However, the BMP-2 + TGF-β1 cocktail and 28 days of culture appears to be the most favourable growing conditions to hinder the expression of type I collagen.

A second western blot experiment was carried out on the same samples but focused particularly on protein expression of type IIB and type IX collagens (Figure 7). This experiment aimed to determine the most favourable growing conditions for maximal expression of these phenotypic markers of hyaline articular cartilage. Thus, the results indicate that for culture conditions in the presence of BMP-2 + TGF-β1 or in the presence of TGF-β3 alone leads to the highest synthesis of type IIB and IX collagens. The expression of these two proteins appeared to be compromised in the presence of BMP-2 + TGF-β3 (Figure 7A). For the second strain studied, our results suggest that growth factors have quite similar effects on the protein expression of type IIB collagen, while the BMP-2 + TGF-β1 cocktail appeared to lead to the synthesis of the highest amounts of type IX collagen, compared with the TGF-β3 and BMP-2 + TGF-β3 cocktail (Figure 7B).

### 2.3. Effect of Long-Term Culture on the Phenotypic Status of the Chondrocytes Derived from BM-MSCs

**Figure 8:**
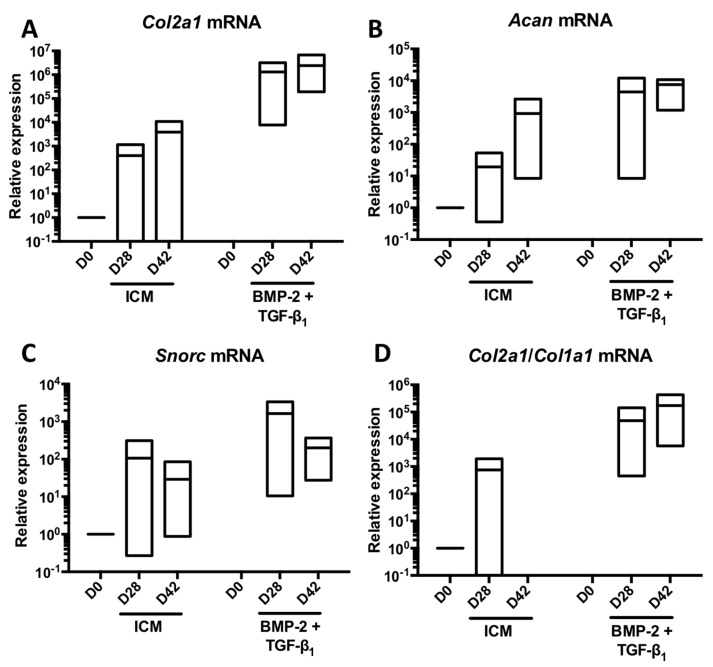
Increase in the culture time of MSCs in the presence of BMP-2 and TGF-β1 to a total of 42 days and its influence on the expression of mRNAs encoding specific markers of hyaline articular cartilage. Equine MSCs derived from bone marrow were amplified and seeded in collagen sponges at P4 (*n* = 3). They were then cultured in hypoxia (3% O_2_) for 28 and 42 days (D28 and D42, respectively) in the presence of incomplete chondrogenic medium (ICM) alone or in association with BMP-2 (50 ng/mL) and TGF-β1 (10 ng/mL) (BMP-2 + TGF-β1). The D0 condition corresponds to the stem cells cultured in monolayer at P4 with the amplification medium. mRNA levels of *Col2a1* (**A**); *Acan* (**B**) and *Snorc* (**C**) were estimated by RT-qPCR after normalization relative to the *β-actin* reference gene. The *Col2a1*:*Col1a1* ratio is shown (**D**). The results are presented as floating bars (mean, extreme values) and the significance of the values was tested using Student’s *t*-test.

**Figure 9:**
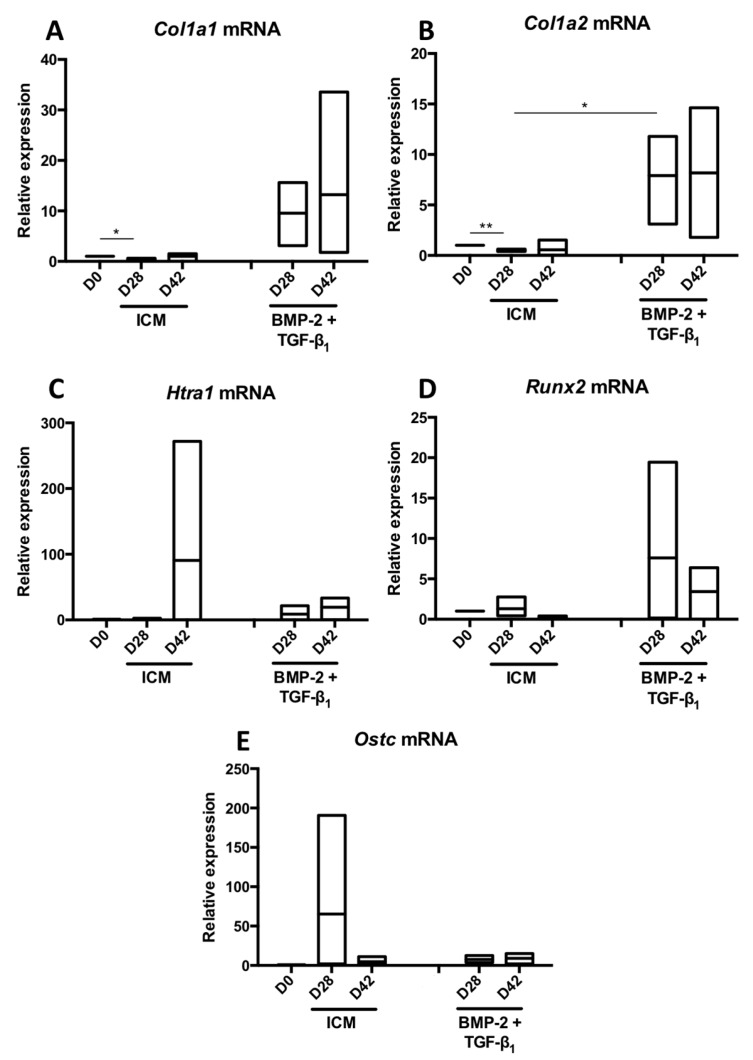
Increase in the culture time of MSCs in the presence of BMP-2 and TGF-β1 to a total of 42 days and its influence on the expression of mRNAs encoding the nonspecific markers of hyaline articular cartilage. Equine MSCs derived from bone marrow were amplified and seeded in collagen sponges at P4 (*n* = 3). They were then cultured in hypoxia (3% O_2_) for 28 and 42 days (D28 and D42, respectively) in the presence of incomplete chondrogenic medium (ICM) alone or supplemented with BMP-2 (50 ng/mL) and TGF-β1 (10 ng/mL) (BMP-2 + TGF-β1). The D0 condition corresponds to the stem cells cultured in monolayer at P4 with the amplification medium. mRNA levels of *Col1a1* (**A**); *Col1a2* (**B**) *Htra1* (**C**); *Runx2* (**D**) and *Ostc* (**E**) were estimated by RT-qPCR after normalization relative to the *β-actin* reference gene. The results are presented as floating bars (mean, extreme values) and the significance of the values was tested using Student’s *t*-test (* *p* < 0.05, ** *p* < 0.01).

Since TGF-β3 alone or in combination with BMP-2 had no additive effect compared with the BMP-2 + TGF-β1 cocktail, to try to improve the chondrocyte phenotype, we tested another hypothesis. Culture was extended in the presence ICM supplemented with or without BMP-2 + TGF-β1 for 42 days to study the behaviour of the MSCs differentiated into chondrocytes in a longer-term culture and compare with a culture of 28 days. Thus, prolonging culture for an additional 14 days on three strains led to a modest increase in the mRNA amounts of *Col2a1* (Figure 8A), *Acan* (Figure 8B) and on the *Col2a1*:*Col1a1* ratio (Figure 8D) compared with a culture time of 28 days. However, the expression of *Snorc* mRNAs dropped dramatically under these conditions (Figure 8C). Regarding the two messengers encoding type I collagen chains, increasing the culture time to 42 days compared with 28 days led to a moderate increase in the *Col1a1* mRNA levels (Figure 9A), whereas those of *Col1a2* remained stable (Figure 9B).

The relative expression of *Htra1* appeared stabilized or slightly increased by extending the culture period in the presence of BMP-2 + TGF-β1 (Figure 9C), while that of *Runx2* was decreased by 2-fold between 28 and 42 days in the presence of these chondrogenic factors (Figure 9D). *Ostc* mRNAs levels did not seem to be influenced by the duration of culture time (Figure 9E).

**Figure 10:**
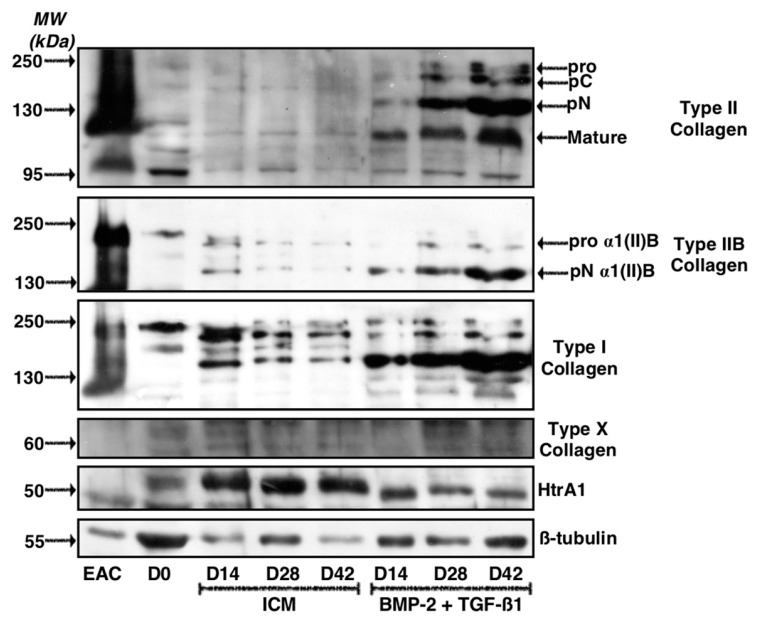
Kinetic study of cartilage matrix proteins expression when MSCs were cultured in the presence of BMP-2 and TGF-β1 for 42 days. Equine MSCs derived from bone marrow were amplified and seeded in collagen sponges at P4. They were then cultured in hypoxia (3% O_2_) for 14, 28 and 42 days (D14, D28 and D42, respectively) in the presence of incomplete chondrogenic medium (ICM) alone or supplemented with BMP-2 (50 ng/mL) and TGF-β1 (10 ng/mL) (BMP-2 + TGF-β1). The sponges were harvested and crushed. The total protein extracts were separated by electrophoresis on a 10% acrylamide gel under denaturing conditions. The proteins were then transferred to a PVDF membrane which was incubated with the anti-collagen II, anti-collagen IIB, anti-collagen I, anti-collagen X, anti-HtrA1 or anti-β-tubulin primary antibody. The molecular weight (MW kDa) is indicated on the left. D0: stem cells cultured in amplification medium at P4; EAC: equine articular cartilage.

The study of ECM protein synthesis in these experimental conditions indicated that a longer incubation period of 42 days led to a strong increase in total type II collagen, including proα1(II)B and pNα1(II)B collagen, characterizing mature chondrocytes (Figure 10). However, the same pattern was observed for type I collagen, which showed high expression in the ECM in these culture conditions, compared with a culture of 14 or 28 days. In the control ICM condition, the expression of type I collagen, however, decreased with culture time, although the expression of β-tubulin was also lower at 14 and 42 days compared with the culture of 28 days. Type X collagen was still not detectable after 42 days of culture, whereas HtrA1 stabilized between 28 and 42 days of culture.

### 2.4. Effect of Knockdown with Col1a1 and/or Col1a2 siRNAs on the Quality of the ECM Synthesized by Chondrocytes Derived from MSCs

**Figure 11:**
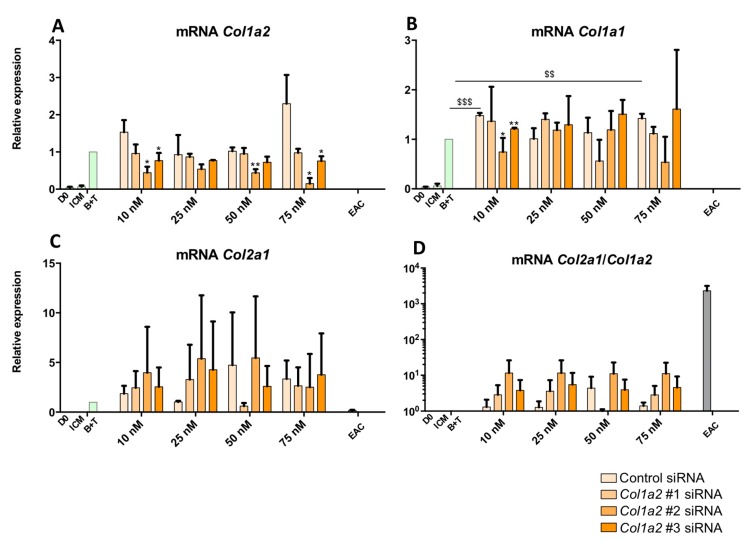
Effect of *Col1a2* siRNAs on the steady-state mRNA amounts of specific and nonspecific markers of chondrocytes derived from MSCs after 14 days of culture. Equine MSCs derived from BM were amplified and seeded in type I/III collagen sponges at P4 (*n* = 2). They were then cultured in hypoxia (3% O_2_) for 14 days in incomplete chondrogenic medium (ICM) in the absence or presence of BMP-2 (50 ng/mL) and TGF-β1 (10 ng/mL) (B + T). The MSCs were transfected with a control siRNA or one of the three *Col1a2* siRNAs tested (#1, #2 and #3, respectively) at various concentrations (10, 25, 50 and 75 nM) in the presence of B + T. The D0 condition corresponds to MSCs cultured in monolayer at P4 with the amplification medium, while the equine articular chondrocyte (EAC) condition corresponds to the mRNAs extracted from EACs obtained from enzymatic digestion of healthy cartilage. mRNA levels of *Col1a2* (**A**); *Col1a1* (**B**) and *Col2a1* (**C**) were estimated by RT-qPCR after normalization relative to the *β-actin* reference gene. The *Col2a1*:*Col1a2* ratio is shown (**D**). A representative experiment is shown. The results present mean values ± SD for the different experimental conditions studied and the significance of the values was tested using Student’s *t*-test to compare : treatment without siRNA (B + T) compared to treatment with a Control siRNA, at each concentration ($ *p* < 0.05, $$ *p* < 0.01, $$$ *p* < 0.001) and treatment with a *Col1a2* siRNA compared to treatment with a Control siRNA, at the same concentration (* *p* < 0.05, ** *p* < 0.01, *** *p* < 0.001).

To attempt to improve the functional index of MSCs differentiated into chondrocytes, an RNA interference approach was implemented. First, the effect of three *Col1a2* siRNAs were tested at the mRNA level to verify their efficiency on the targeted mRNAs (Figure 11). The cells were incubated for 14 days. The results show that transfection with a control siRNA had no significant effect on the *Col1a2* mRNAs, except at a concentration of 75 nM where off-target action appeared, causing an increase in levels (Figure 11A). Regarding the effect of the tested *Col1a2* siRNAs, the *Col1a2* siRNA #1 did not cause any significant effect on the expression of *Col1a2* mRNAs, unlike the *Col1a2* siRNA #2 which led to a significant decrease of about 50% of the expression of *Col1a2* messengers compared with the control siRNA condition at the same concentration. Transfection of 75 nM of *Col1a2* siRNA #2 caused stronger inhibition of its target, although an off-target effect was observed upon transfection of a control siRNA at the same concentration. Regarding *Col1a2* siRNA #3, although there was a knockdown trend in the amounts of *Col1a2* mRNAs, the effects were very weak and *Col1a2* siRNA #2 remained the most effective on its target.

*Col1a1* mRNA analysis indicated that transfection with *Col1a2* siRNA does not modulate its expression (Figure 11B). Furthermore, there was no significant effect when the cells were transfected with 25 and 50 nM of siRNAs, although off-target effects or an indirect effect via the production of other proteins caused by the silencing of *Col1a2* were observable with 75 nM of *Col1a2 #3* siRNAs. Concerning *Col2a1* mRNA (Figure 11C), although there was some inter-strain variability, transfection by different siRNAs led to no significant modulation of its expression. Finally, analysis of the *Col2a1*:*Col1a2* ratio indicates that transfection with *Col1a2* siRNA #2 led to an increase in this ratio, regardless of the concentration (Figure 11D). This ratio was enhanced by 10-fold compared with the B + T condition, although it remained lower than that found in EACs, due to persistent expression of *Col1a2* mRNA in the MSC culture. There was no *Col1a2* expression in EACs (Figure 11A).

**Figure 12:**
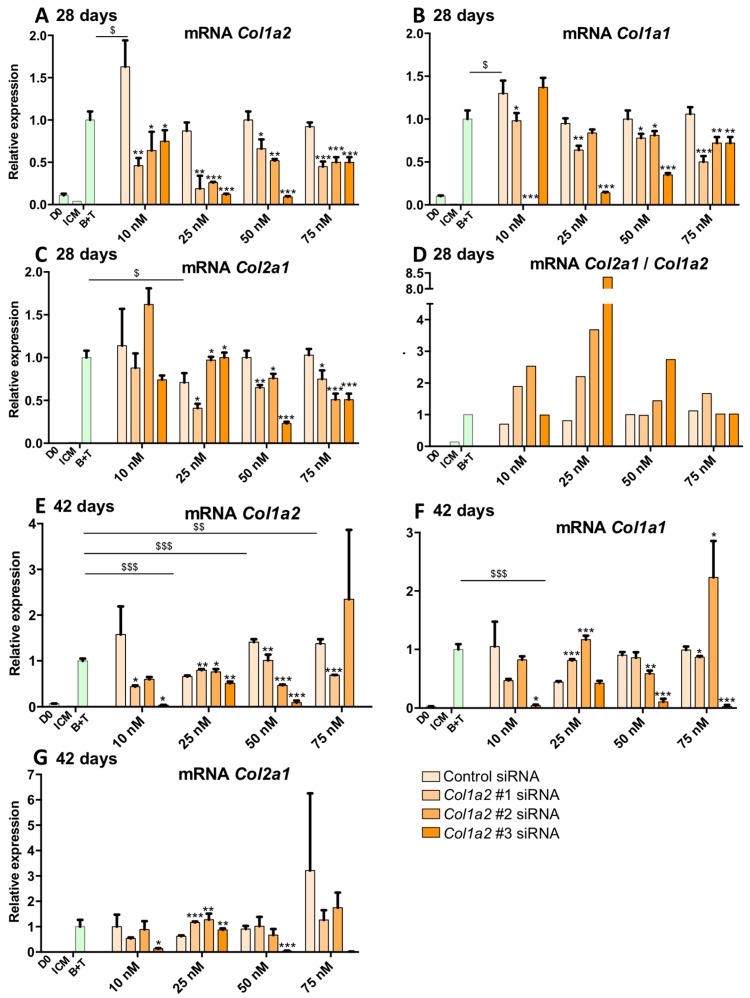
Effect of *Col1a2* siRNAs on the mRNA amounts of specific and nonspecific markers of chondrocytes derived from MSCs cultured for 28 and 42 days. Equine MSCs derived from BM are amplified and seeded in type I/III collagen sponges at P4 (*n* = 3, one of three representative experiments is shown). They were then cultured in hypoxia (3% O_2_) for 28 days (**A**–**D**) or 42 days (**E**–**G**) in incomplete chondrogenic medium (ICM) in the absence or presence of BMP-2 (50 ng/mL) and TGF-β1 (10 ng/mL) (B + T). The MSCs were transfected with a control siRNA or one of the three *Col1a2* siRNAs tested (#1, #2 and #3, respectively) at various concentrations (10, 25, 50 and 75 nM) in the presence of B + T. The D0 condition corresponds to MSCs cultured in monolayer at P4 with the amplification medium. mRNA levels of *Col1a2* (**A**,**E**), *Col1a1* (**B**,**F**) and *Col2a1* (**C**,**G**) were estimated by RT-qPCR after normalization relative to the *β-actin* reference gene. The *Col2a1*:*Col1a2* ratio is shown (**D**). The results present mean values ± SD for the different experimental conditions studied and the significance of the values was tested using Student’s *t*-test to compare : treatment without siRNA (B + T) compared to treatment with a Control siRNA, at each concentration ($ *p* < 0.05, $$ *p* < 0.01, $$$ *p* < 0.001) and treatment with a *Col1a2* siRNA compared to treatment with a Control siRNA, at the same concentration (* *p* < 0.05, ** *p* < 0.01, *** *p* < 0.001).

As described above, increasing the culture time to 28 and 42 days led to a significant enhancement in the expression of the markers specific to hyaline articular cartilage. However, in parallel, the expression of type I collagen also increased markedly. To verify the validity of the approach of RNA interference to increase the functional index of MSCs differentiated into chondrocytes, we also tested the effect of our *Col1a2* siRNAs at 28 days (Figure 12A–D) and 42 days of culture (Figure 12E–G). A representative experiment of three replicates is presented. Our results indicate that the *Col1a2* mRNAs levels were systematically reduced by transfection of *Col1a2* siRNAs compared to the control siRNA condition at the same concentration (Figure 12A). This effect is most pronounced at a concentration of 25 nM for all *Col1a2* siRNAs and 25 and 50 nM when considering the *Col1a2* siRNA #3. Inhibition with the *Col1a2* siRNA #3 transfected at a concentration of 25 and 50 nM was approximately 80% compared with the control siRNA condition at the same concentrations. Of interest, this transfection with *Col1a2* siRNAs seemed to lead to a moderate decrease in the expression of the *Col1a1* (Figure 12B). This inhibition was most pronounced for the *Col1a2* siRNA #3 at 25 and 50 nM (70% and 50% respectively) compared with the control siRNA condition at identical concentrations. These results suggest that the *Col1a2* siRNA #3 targets the mRNAs encoding the two chains of type I collagen. Regarding the *Col2a1* mRNAs, transfection with 25 nM of *Col1a2* siRNA #1 led to a decrease of 50% of its amount compared with the control siRNA condition at the same concentration (Figure 12C). Transfection with *Col1a2* siRNAs #2 or #3 did not influence its expression compared with the B + T condition, whereas at 50 nM, the *Col1a2* siRNA #3 caused an off-target effect leading to a significant decrease in *Col2a1* mRNA levels. Transfection at a molarity of 75 nM showed off-target effects, involving *Col1a2* siRNA #2 and #3 in particular, compared with control siRNA at the same concentration, or in the B + T cocktail. Finally, the *Col2a1*:*Col1a2* ratio remained unchanged when MSCs were transfected with a control siRNA, compared with the B + T condition (and no siRNA) and regardless of the transfection concentration (Figure 12D). The ratio increased 2-fold when cells were transfected with *Col1a2* siRNA #1 at 10 nM and 25 nM, while transfection of higher concentrations did not affect this ratio compared with the control siRNAs conditions. These same trends were slightly amplified upon transfection with the *Col1a2* siRNA #2, while the *Col1a2* siRNA #3 caused an increase in this ratio by a factor of 8.5 at 25 nM and 3 at 50 nM.

When the cells were cultured for 42 days, transfection with *Col1a2* siRNA #3 (10 and 50 nM) led to a highly significant inhibition of the expression of *Col1a2* mRNAs (Figure 12E). Moreover, 50 nM of *Col1a2* siRNAs #2 caused a 50% decrease in the expression of the target compared to the control siRNA condition. Furthermore, transfection with *Col1a2* siRNAs #3 invariably led to a very strong inhibition of the expression of *Col1a1* mRNAs (Figure 12F), whereas the *Col1a2* siRNA #1 did not appear to have a significant effect and *Col1a2* siRNA #2 caused a modest decrease in the expression of *Col1a1* compared with control siRNA and with the B + T cocktail alone (no siRNA). At 75 nM, *Col1a2* siRNA #2 caused off-target effects and led to increased expression of *Col1a1* mRNAs. Moreover, with regard to the expression of *Col2a1* mRNAs, *Col1a2* siRNA #3 caused a collapse in its expression, suggesting a strong off-target effect (Figure 12G), whereas none of the other conditions modulate the expression of *Col2a1*.

All these results obtained after 14, 28 and 42 days of culture indicate that *Col1a2* siRNA #2 is the best candidate for the RNA interference strategy. This siRNA species decreased *Col1a2* expression, albeit sometimes more modestly than *Col1a2* siRNA #3, with much less marked off-target effects.

**Figure 13:**
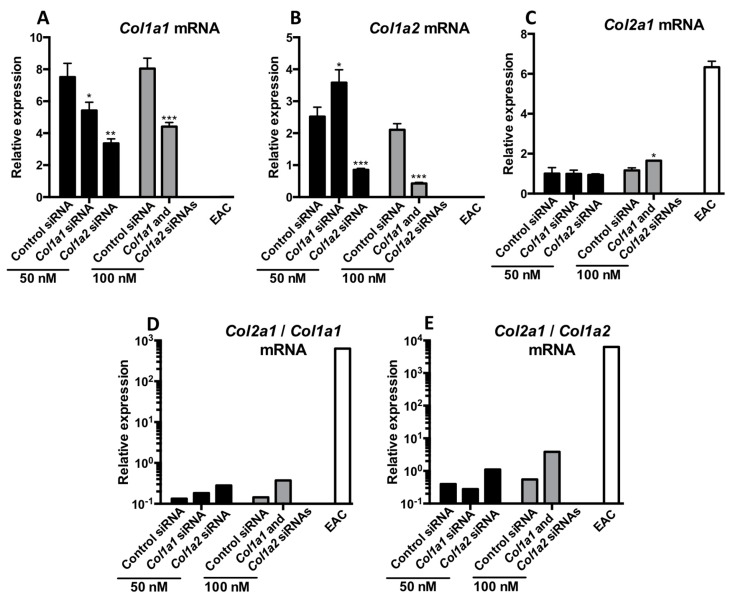
Effect of *Col1a1* and/or *Col1a2* siRNAs on the mRNA levels of specific and nonspecific markers of chondrocytes derived from MSCs cultured for 42 days. Equine MSCs derived from BM were amplified and seeded in type I/III collagen sponges at P4 (*n* = 3, one of three representative experiments is shown). They were then cultured in hypoxia (3% O_2_) for 42 days (**A**–**E**,**G**) in incomplete chondrogenic medium (ICM) in the presence of BMP-2 (50 ng/mL) and TGF-β1 (10 ng/mL). The MSCs were transfected with a control siRNA or *Col1a1* and/or *Col1a2* siRNAs (50 nM of each siRNA was transfected). After 42 days of culture, mRNA levels of *Col1a1* (**A**); *Col1a2* (**B**) and *Col2a1* (**C**) were estimated by RT-qPCR after normalization relative to the *β-actin* reference gene. The *Col2a1*:*Col1a1* and *Col2a1*:*Col1a2* ratios are shown (**D**,**E** respectively). The results present mean values ± SD for the different experimental conditions studied and the significance of the values was tested using Student’s *t*-test (* *p* < 0.05, ** *p* < 0.01, *** *p* < 0.001).

**Figure 14:**
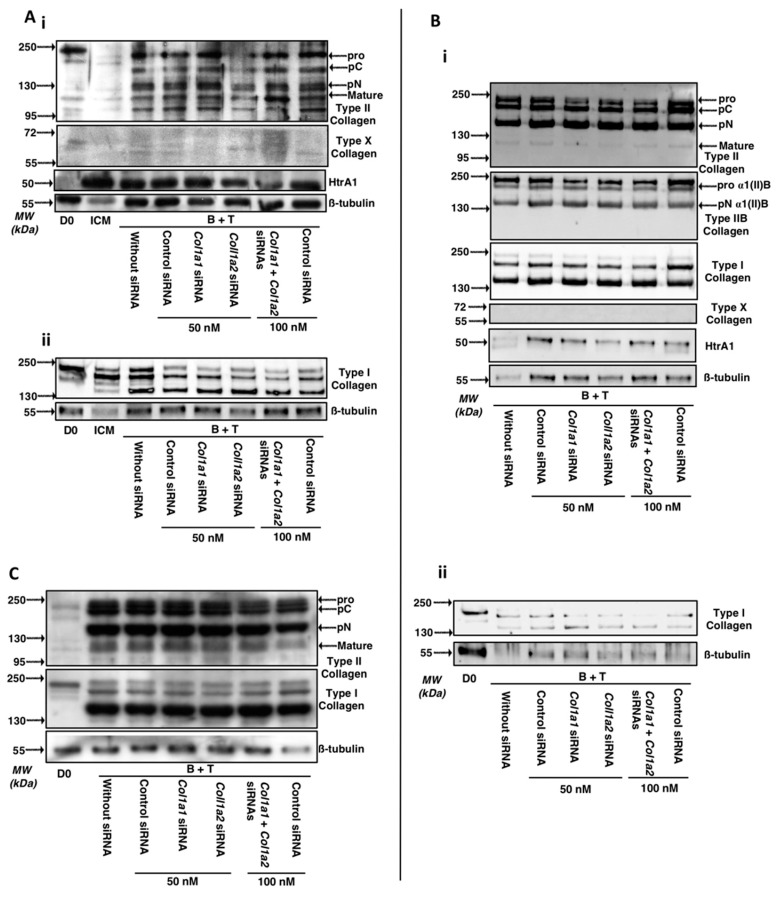
Effect of *Col1a1* and/or *Col1a2* siRNAs on the protein levels of specific and nonspecific markers of chondrocytes derived from MSCs cultured for 14, 28 and 42 days. Equine MSCs derived from BM were amplified and seeded in type I/III collagen sponges at P4 (*n* = 3, one of three representative experiments is shown). They were then cultured in hypoxia (3% O_2_) for 14, 28 and 42 days (**A**–**C** respectively) in incomplete chondrogenic medium (ICM) in the presence of BMP-2 (50 ng/mL) and TGF-β1 (10 ng/mL). The MSCs were transfected with a control siRNA or *Col1a1* and/or *Col1a2* siRNAs (50 nM of each siRNA was transfected). After 14, 28 and 42 days of culture, the total protein extracted (15 µg for panels **A** and **C**; 5 µg (panel **Bi**) and 2.5 µg (**Bii**)) from type I/III collagen sponges were separated by electrophoresis on a 10% acrylamide gel under denaturing conditions. Panels **Ai** (type II and X collagens and HtrA1) and **Aii** (type I collagen) represent western-blots obtained from two strains. The proteins were then transferred to a PVDF membrane and the latter was incubated with the anti-collagen II, anti-collagen I, anti-collagen X, anti-HtrA1 or anti-β-tubulin primary antibody. The molecular weight marker (MW, kDa) is indicated on the left of the panels. D0: stem cells grown in amplification medium; ICM: MSCs cultured in ICM medium without growth factors.

Finally, to check whether simultaneous knockdown of both *Col1a1* and *Col1a2* siRNA is a more appropriate strategy to increase the quality of newly synthesized cartilage, simultaneous transfection of *Col1a1* siRNA (previously tested and validated) and *Col1a2* siRNA (*Col1a2* siRNA #2 of the present study) was carried out after incubation of the MSCs treated with the B + T cocktail for 42 days and then RT-qPCR (Figure 13A–E) and western blot were performed (Figure 14). These siRNAs were transfected individually or in combination (50 nM each).

*Col1a1* siRNA caused a 30% decrease in the mRNA amounts of its target (Figure 13A) compared with the control siRNA condition, whereas the *Col1a2* siRNA caused a 50% knockdown of *Col1a1* mRNAs%. Simultaneous transfection of the two siRNAs induced the same 50% inhibition in *Col1a1* mRNA expression. Similarly, transfection with the *Col1a2* siRNA alone or in association with *Col1a1* siRNA induced a decrease of approximately 50% in the expression of *Col1a2* (Figure 13B). None of the experimental conditions studied here influenced the expression of *Col2a1* (Figure 13C). Finally, the *Col2a1*:*Col1a1* (Figure 13D) or the *Col2a1*:*Col1a2* ratios (Figure 13E) were highest when the cells were transfected with *Col1a2* siRNA alone or combination with *Col1a1* siRNA, even though this ratio was lower compared to that observed in EACs.

Regarding the ECM proteins secreted by MSCs differentiated into chondrocytes (Figure 14A), the results of RT-qPCR experiments confirmed the results of the 14-day cultures: the various maturation forms of type II collagen were not modulated by the siRNAs, with the possible exception of the *Col1a2* siRNA. Thus, type II collagen seems less quantitatively present in the ECM after 14 days in the *Col1a2* siRNA treated group.

Regarding type I collagen after 14 days of culture, transfection with the *Col1a1 and Col1a2* siRNAs prevents its expression, especially the immature forms which were less detectable in neocartilage ECM, while the mature form of type I collagen appeared to be not down-regulated (Figure S2, for the quantitative analysis of the membranes). All these culture conditions with the siRNA treatment did not affect the expression of HtrA1 or type X collagen, the latter being undetectable under these conditions. Almost similar data upon transfection of *Col1a1* and/or *Col1a2* siRNAs are observed after 28 days of culture on types I, II and X collagens as well as HtrA1 (Figure 14B, the quantitative analysis of types II and I membranes are presented in Figure S3). The best combination to decrease all maturation forms of type I collagen is observed upon *Col1a1* and *Col1a2* siRNAs co-transfection, even though the *Col1a2* siRNA alone had a more pronounced interference effect compared to the *Col1a1* siRNA (Figure 14 B(ii)).

Different results are obtained during the study of matrix proteins after 42 days of culture (Figure 14C). Indeed, in these culture conditions, the protein levels of type II collagen are not significantly modulated by transfection of siRNAs (regardless of the siRNA considered), compared to a culture of 42 days with B + T without transfection of siRNAs. The protein levels of the mature form of type I collagen is not modulated by transfection of *Col1a1* and/or *Col1a2* siRNAs compared to the control siRNA condition (50 nM). In contrast, transfection with the *Col1a2* siRNA in combination with a *Col1a1* siRNA appears to modestly decrease the protein levels of immature forms of type I collagen (Figure S4, quantitative analysis of type I collagen membranes). *Col1a1* siRNA has no effect on the matrix expression of type I collagen, compared to the control siRNA condition.

## 3. Discussion

The aim of this study was to improve the protocol of chondrogenic differentiation of equine BM-MSCs. To date, the best protocol in our hands calls for culturing the cells in hypoxia in type I/III collagen sponges in the presence of BMP-2 and TGF-β1 for 14 days. Here, we studied the effect of TGF-β3 as a chondrogenic factor instead of the BMP-2 + TGF-β1 combination and we increased culture time. Hypoxia has been proven time again beneficial for maintaining viability, proliferation and cell migration [34,35] and more specifically for promoting the differentiation of MSCs into chondrocytes [36]. Moreover, hypoxia blocks differentiation at the mature chondrocyte stage, avoiding chondrocyte hypertrophy [37,38]. Similarly, although there are other suitable biomaterials (e.g., type II collagen sponges) for cartilage engineering approaches, we chose type I/III collagen sponges since various studies have shown that chondrocytes and MSCs can be cultured in this scaffold [24,25]. Fibrin sealants are used to insert the neocartilage constructs in the joint and sutures can be added if needed. Furthermore, these sponges do not subsist in vivo since animal studies have shown that about 50% of the scaffold is degraded after 2 weeks [39].

Increasing the culture time to 28 days led to an increase in the markers specific to hyaline articular cartilage. However, type I collagen expression also increased but the level of expression of HtrA1 was lower than in the conventional 14 days culture. In addition, the increase in the specific markers of the chondrocyte phenotype with the BMP-2 and TGF-β1 cocktail appears to be strain-dependent and in some strains, synthesis of type II collagen was maximal after only 14 days of culture. In these strains, protein synthesis of type IX collagen was also maximal after 14 days of culture and no longer varied with culture time. These results are in agreement with the literature data. In general, experts recommend maintaining a culture for at least 21 days to assay the chondrogenic differentiation potential of MSCs [40]. However, other studies suggest that a culture of 28 days increases the amount of glycosaminoglycans present in cartilage ECM but does not significantly influence type II collagen and aggrecan expression. Additionally, in vitro culture time extended to 28 days does not prevent endochondral ossification in vivo following subcutaneous implantation of the cartilaginous substitute [41]. We even extended culture time to 6 weeks to study the fate of the phenotypic markers of articular cartilage at a longer term and the same patterns as those observed after 28 days were observed. However, the expression of type I collagen also increased and it is difficult to determine the exact culture time required to obtain an optimal type II:type I collagen ratio at the protein level. Qualitative studies, i.e., immunohistochemical studies, are necessary to assess the quality of the neocartilage synthesized more accurately. Moreover, *Snorc* mRNA amounts collapsed after 42 days of culture. This transmembrane proteoglycan is co-expressed with *Col2a1*, *Acan* and *Sox9* [5] and the decrease in its expression may suggest an earlier regulation than other chondrocyte-specific markers when the optimal mature chondrocyte phenotype is acquired. In this case, longer in vitro culture times may be detrimental for the fate of the neocartilage after implantation. Therefore, a culture time of 14 to 28 days for chondrogenic differentiation prior to in vivo implantation is likely more appropriate. One study has shown that for longer culture times (up to 112 days; i.e., 16 weeks), protein expression of type II collagen is lower than in cultures of 56 days (8 weeks) and that of type I collagen is higher. Moreover, cell viability decreases dramatically after 56 days of culture compared with 28 days and literally collapses after 112 days of culture, with cell viability of less than 20% [42]. Taken together, our data suggest that culture times of 14 to 28 days for the neocartilage constructs intended for implantation appears to be the best compromise between ECM synthesis, ECM quality and cost-effectiveness.

Our study also attempted to determine whether a culture in the presence of TGF-β3 is more beneficial than TGF-β1, which is conventionally used in our protocol. Both isoforms are used to induce chondrogenesis of MSCs [28,43]. Our study shows that the use of TGF-β3 in the differentiation of equine BM-MSCs led to higher synthesis of type II collagen protein compared with the BMP-2 + TGF-β1 cocktail. However, type I collagen protein synthesis was once again amplified without a concomitant decrease in HtrA1 expression, which even increased relative to BMP-2 + TGF-β1 or BMP-2 + TGF-β3 cocktails. Regarding the mRNAs encoding the different markers studied, there was no advantage in the use of TGF-β3 in terms of either specific or non-characteristic markers of hyaline articular cartilage (i.e., dedifferentiation markers, hypertrophy or terminal differentiation). Our results confirm our initial hypothesis, namely, that the BMP-2 + TGF-β1 cocktail provides for the synthesis of a cartilage substitute the most similar to the hyaline articular cartilage of healthy horses.

Nonetheless, regardless of the culture conditions considered and regardless of the length of culture time, undesirable expression of type I collagen remains. Therefore, to try to improve the functional index of chondrocytes derived from equine MSCs, we further explored the benefits of using siRNAs targeting *Col1a1* and *Htra1* [27]. Our results have shown 50% inhibition of *Col1a1* mRNAs as well as for *Htra1*. Moreover, the use of BMP-2 + TGF-β1 to differentiate equine MSCs led to a large decrease in HtrA1 protein expression, unlike for human chondrogenic MSCs differentiation [25,44], suggesting that RNA interference targeting HtrA1 is not necessary for equine cells. We conclude that the development and improvement of our strategy to reduce the high residual expression of type I collagen was justified but not for HtrA1.

RNA interference strategies using new siRNAs targeting other regions of *Col1a1* mRNA were tested. We also evaluated knocking down *Col1a2* instead of *Col1a1*, or both simultaneously. An imbalance in the stoichiometry of the presence of mRNAs encoding the two chains of type I collagen, which should theoretically be present at the ratio of two chains α1(I) for one chain α2(I) is expected to lead to degradation of the mRNA species present in excess as well as protein complexes [45,46,47]. The knockdown effect appeared more efficient when targeting *Col1a2* mRNA compared with an RNA interference strategy targeting *Col1a1* mRNAs. Type I collagen protein expression nevertheless persisted but practically met the same level of effectiveness found in the strategies developed in the human model, particularly when using in vitro amplified chondrocytes [26]. In addition, the development of this strategy in MSCs remains complex: it is difficult to obtain results perfectly equivalent to those obtained with dedifferentiated chondrocytes, because type I collagen is constitutively expressed and in very high amounts in MSCs [48]. However, it seems that our interference effect is weakly more pronounced when both *Col1a1* and *Col1a2* siRNAs were co-transfected. Nevertheless, it appears that the synthesis of type I collagen is weakly modulated by siRNAs after 42 days of culture. These results suggest that the synthesis of too high amounts of ECM no more allows siRNAs to block protein synthesis of type I collagen. This could be explained by the fact that dense ECM prevents the intracellular distribution of siRNAs or that transfection does not prevent the progressive accumulation of type I collagen during culture time. Therefore, this siRNA approach, which also shows better knockdown effects, seems more appropriate to limit the production of type I collagen at a shorter incubation time of the cultures, typically 14 and 28 days, before the emergence of too high amounts of ECM. Considering these results, it would be more appropriate to modify the intracellular distribution strategy of siRNAs. For example, nanocarriers have been tested for the delivery of siRNAs, particularly in the context of cancer research, to target multidrug-resistant cancers [49]. In addition, to reduce the potential toxicity of siRNA transfection, there are various types of nanocarriers (natural or artificial) and they can be developed to fit with the proposed strategy. For example, quantum dots combined with *RUNX2* siRNA by electrostatic interactions have been developed recently to reduce the hypertrophy of MSCs differentiated into chondrocytes [50]. This type of approach, although complex, opens up many opportunities to overcome the persistent expression of type I collagen.

All these data indicate that the original cocktail of BMP-2 + TGF-β1 is the best solution for the chondrogenic differentiation of equine BM-MSCs, despite the higher expression of type II collagen matrix when TGF-β3 is used. The cartilage ECM components also showed enhanced expression for culture times of up to 6 weeks. However, the increase in ECM production was moderate after 14 days of culture and the best compromise between high ECM synthesis and minimal production of type I collagen still needs to be identified. Thus, culture times between 14 and 28 days remains the best compromise.

Before considering in vivo trials in an equine clinical setting, the knockdown strategy should be improved. However, to date, our differentiation protocol may be applicable for pre-clinical trials in the horse to help to decide on the best in vitro culture conditions to produce hyaline-type articular cartilage in vivo.

## 4. Materials and Methods 

### 4.1. Cell Isolation and Cell Culture

All samples of bone marrow and cartilage were obtained at the Centre d’Imagerie et de Recherché sur les Affections Locomotrices Equines (CIRALE, Goustranville, France). Equine BM was collected from sternal puncture in a cohort of horses whose age ranged from 2 to 4 years. The volume of bone marrow collected was from 30 to 40 mL. The BM-MSCs samples were collected into sterile flasks containing 40 mL of citrate phosphate dextrose anticoagulant, stored at room temperature and processed within 1 to 2 h after collection. To isolate mononuclear cells (MNCs), each MSCs unit was diluted 1:1 with phosphate-buffered saline (PBS, Invitrogen Life Technologies, Carlsbad, CA, USA) and carefully mixed with Ficoll-Paque PREMIUM (GE Healthcare Bio-Sciences, Chicago, IL, USA) medium. After density gradient centrifugation at 400× *g* for 30 min at room temperature, MNCs were washed once with PBS. MSCs-derived MNCs were seeded in culture flasks in low glucose-Dulbecco’s modified Eagle Medium (LG-DMEM, Invitrogen Life Technologies, Carlsbad, CA, USA) containing 30% foetal calf serum (FCS, Invitrogen Life Technologies, Carlsbad, CA, USA), 10^−7^ M dexamethasone (Sigma-Aldrich, St. Louis, MO, USA) and incubated at 37 °C in a 5% CO_2_ atmosphere. A cocktail of antimicrobials composed of 100 IU/mL of penicillin, 100 µg/mL of erythromycin and 0.25 mg/mL of fungizone was added to all the media used in this study. Non-adherent cells were removed 24 h after initial plating. The medium was changed twice weekly until adherent cells appeared, defined as passage zero (P0). After the appearance of several colonies in samples, cells were detached using trypsin/EDTA (Invitrogen Life Technologies, Carlsbad, CA, USA) and then reseeded at 5000 cells/cm^2^ (passage one, P1). Cell expansion was performed in the LG-DMEM media containing 20% FCS. The culture medium was changed three times per week and cells were passaged at 80% confluency until passage 3 (P3).

All the BM-MSCs strains used in the present study have been previously characterized. Thus, these BM-MSCs present a very high proliferation potential and these cells satisfy the criteria of stem cells definition established by the International Society for Cellular Therapy (ISCT) since they have the ability to adhere to a plastic culture support, they express a panel of characteristic surface proteins and possess multipotency capacity to differentiate into osteoblasts, adipocytes and chondrocytes [27].

Equine articular chondrocytes (EAC) were prepared from healthy metacarpal joint. Cartilage samples were cut into small slices, then chondrocytes were isolated by sequential digestion for 45 min at 37 °C with 2 mg/mL of type XIV protease (Sigma-Aldrich, St. Louis, MO, USA) and then overnight at 37 °C with 1 mg/mL of type I collagenase (from *Clostridium histolyticum*; Invitrogen Life Technologies, Carlsbad, CA, USA). The cell suspension was filtered through a 70 μm mesh nylon membrane and centrifuged at 200× *g* for 10 min. The pelleted cells were re-suspended in Trizol (Invitrogen) and RNA extraction was carried out according to the manufacturer’s protocol. For western blot experiments, small cartilage slices were ground in liquid nitrogen and protein extraction was performed with RIPA buffer. EAC extracts were used in real-time reverse transcription-polymerase chain reaction (RT-PCR) and western blots as controls.

### 4.2. Chondrogenic Differentiation in 3D

The scaffold was manufactured by Symatèse Biomatériaux (Chaponost, France). These collagen sponges (100% of collagen, 2 mm thickness, 5 mm diameter, corresponding to a volume of 0.04 cm^3^, around 100 µm pore size) are composed of native type I (90–95%) and type III (5–10%) collagens from calf skin; they are crosslinked with glutaraldehyde to increase their stability. They are sterilized with β-radiation and they do not swell after rehydration.

MSCs were grown for 7, 14, 21, 28 and 42 days in a 3D scaffold to induce chondrogenesis according to the following protocol. Briefly, cells were subcultured as monolayers until P3, trypsinized and suspended in Incomplete Chondrogenic Medium (ICM, composed of high glucose-DMEM (HG-DMEM), dexamethasone 10^−7^ M, ascorbic acid-2-phosphate (50 μg/mL), proline (40 μg/mL, Merck, Darmstadt, Germany), sodium pyruvate (1 nM, Invitrogen Life Technologies, Carlsbad, CA, USA), Insulin Transferrin Selenium (ITS, diluted 1%, Sigma-Aldrich, St. Louis, MO, USA). Cell seeding onto the collagen sponges was performed by dropping 20 μL of the cell suspension on the sponge (5 × 10^5^ cells/sponge) in 96-well culture plates and incubating the plates at 37 °C under 5% CO_2_. After 1 h, the cell constructs were transferred to 24-well plates in hypoxia (3% O_2_), in ICM with BMP-2 (Inductos, 50 ng/mL, 12 mg dibotermin alpha, Medtronic BioPharma B.V., Dublin, Ireland) and TGF-β1 (10 ng/mL, Miltenyi Biotec, Bergisch Gladbach, Germany) or with TGF-β3 (10 ng/mL, Bio-Techne, Minneapolis, Minn, USA) or with BMP-2 (50 ng/mL) and TGF-β3 (10 ng/mL). This medium was changed twice a week until the end of the culture.

MSCs monolayers before induction were used as controls (day zero, D0).

### 4.3. RNA Interference-Knockdown of Col1a1 and/or Col1a2 mRNAs

When transfections were performed, MSCs were transfected with a mix of INTERFERin (6 μL/mL, Polyplus-transfection SA, Illkirch-Graffenstaden, France), OptiMEM (20% of the total volume of medium, Invitrogen Life Technologies, Carlsbad, CA, USA) and siRNAs for 10 min at room temperature. siRNAs were used at 10, 25, 50, 75 and 100 nM. Then, the INTERFERin-siRNA complexes were added to the culture medium in which are present the collagen sponges seeded with the cells. When the culture performed for 14 days, the siRNAs are added to the medium at D0 and D7. When the culture was done for 28 days, the siRNAs were added to the medium after a chondrogenic differentiation phase without transfection, at D14, D17, D21 and D24. During a culture of 42 days, the same kinetics as for 28 days was adopted except that a final transfection was realized at D35.

siRNAs specifically targeted the *Col1a1* mRNA (siRNA sequence: 5′-GACAGUGAUCGAAUACAAA-3′, Eurogentec, Liège, Belgium) and the *Col1a2* mRNA (siRNA sequence: n°1, 5′-GAUGGCUGCUCUAGAAAGA-3′; n°2, 5′-GCCAAGAACUGGUACAGAA-3′; n°3, 5′-GUUGACGCUACUCUGAAAU-3′, Eurogentec, Liège, Belgium). A control siRNA was equally used (siRNA sequence: 5′-UUCUCCGAACGUGUCACGU-3,’ Eurogentec, Liège, Belgium).

### 4.4. RNA Isolation and RT-qPCR

After treatment, sponges seeded with cells were rinsed once with ice-cold phosphate-buffered saline and total RNA was extracted using Trizol Reagent according to manufacturer’s instructions. One microgram of RNA was reverse transcribed into cDNA using reverse transcriptase (MMLV, Invitrogen) and oligodT (Eurogentec). PCR was performed on a StepOnePlus Real-Time PCR Systems using Power SYBR Green PCR (Applied Biosystems, Foster City, CA, USA). Sequences of the primers used are listed in Table 1. Relative gene expression was calculated using the 2^−ΔΔCT^ method and expressed as the mean of triplicate samples. Each sample was normalized to *β-actin*.

### 4.5. Western Blots

After treatment, sponge constructs containing cells were rinsed once with ice-cold PBS, crushed and total proteins were extracted using the RIPA-lysis buffer with a protease inhibitor cocktail. Protein concentration was assessed according to the Bradford colorimetric procedure (Bio-Rad, Hercules, CA, USA). Then, 15 µg of total proteins (if not specified) were separated in 10% polyacrylamide gels containing 0.1% SDS and transferred to a polyvinylidene difluoride membrane (PVDF, Merck Millipore, Billerica, MA, USA). Unspecific binding sites of the membranes were blocked with 10% non-fat milk powder in Tris-buffered saline with 0.1% Tween (TBST) for 2 h. Then, membranes were incubated overnight at 4 °C with rabbit anti-human type I collagen (Novotec, Bron, France), rabbit anti-human type II collagen (Novotec, Bron, France), rabbit anti-human type IIB collagen (Covalab, Villeurbanne, France), mouse anti-human type X collagen (Sigma-Aldrich, St. Louis, MO, USA), rabbit anti-human HtrA1 (Merck Millipore, Billerica, MA, USA) or rabbit anti-human β-tubulin (Santa Cruz Biotechnology, Dallas, TX, USA). Concerning the anti-human type IIB collagen, its generation was made by Covalab using exactly the same strategy as the one used by Aubert-Foucher et al. [51]. This antibody detects the pro α1(II)B and pN α1(II)B isoforms of type II collagen, according to the epitope targeted. The following day, membranes were washed three times, followed by an incubation with HRP-conjugated goat anti-rabbit or mouse IgG antibody (Jackson Immunoresearch, West Grove, PA, USA). Signals were visualized with the chemiluminescence method (ECL plus western blotting detection reagent+, Santa Cruz Biotechnology, Inc., Dallas, TX, USA) and developed on X-ray film.

### 4.6. Statistical Analysis

All experiments were repeated at least two times with cells from different horses. Values are reported as means ± SD, floating bar graphs, or box plots. Statistical analyses were performed using the Mann-Whitney U-test or the Student’s *t*-test to determine significant differences between two groups. Statistical analyses were done using Prism (Graphpad, San Diego, CA, USA) or Excel 2016 for Mac (Microsoft, Redmond, DC, USA). A *p*-value of ≤0.05 was considered to be significant.

## Figures and Tables

**Table 1:** Primers used for RT-qPCR experiments.

Gene	Primer Sequence (5′-3′) (F: Foward; R: Reverse)
*β-actin*	F: AGGCACCAGGGCGTGAT
	R: CTCTTGCTCTGGGCCTCGT
*Acan*	F: TGTCAACAACAATGCCCAAGAC
	R: CTTCTTCCGCCCAAAGGTCC
*Col1a1*	F: TGCCGTGACCTCAAGATGTG
	R: CGTCTCCATGTTGCAGAAGA
*Col1a2*	F: CCAGAGTGGAGCAGCGGTTA
	R: GGGATGTTTTCAGGTTGAGCC
*Col2a1*	F: GGCAATAGCAGGTTCACGTACA
	R: CGATAACAGTCTTGCCCCACTT
*Col9a1*	F: CCAAGAGGCCCAATCGACAT
	R: GGGGAAGTCCGTTATCCTGG
*Col10a1*	F: GCACCCCAGTAATGTACACCTATG
	R: GAGCCACACCTGGTCATTTTC
*Col11a1*	F: TTGCTGATGGGAAGTGGCAT
	R: GCTGCTTTGGGGTCACCTAT
*Htra1*	F: GGACTTCATGTTTCCCTCAA
	R: GTTCTGCTGAACAAGCAACA
*Ostc*	F: AGAGTCTGGCAGAGGTGCAG
	R: TCGTCACAGTCTGGGTTGAG
*Runx-2*	F: GCAGTTCCCAAGCATTTCAT
	R: CACTCTGGCTTTGGGAAGAG
*Snorc*	F: TTTACCAGCTCAGTCCTCGG
	R: CAGACAGAGAGCCATCCTGG

## Supplementary Materials

Supplementary materials can be found at http://www.mdpi.com/1422-0067/19/2/435/s1.
