# Supplementary material for: Improvement of the Chondrocyte-Specific Phenotype upon Equine Bone Marrow Mesenchymal Stem Cell Differentiation: Influence of Culture Time, Transforming Growth Factors and Type I Collagen siRNAs on the Differentiation Index
Source: Int J Mol Sci. 2018 Feb 1;19(2):435. doi: 10.3390/ijms19020435 (PMC5855657; doi:10.3390/ijms19020435)
Supplement: Supplementary file 1 [file ijms-19-00435-s001.pdf]

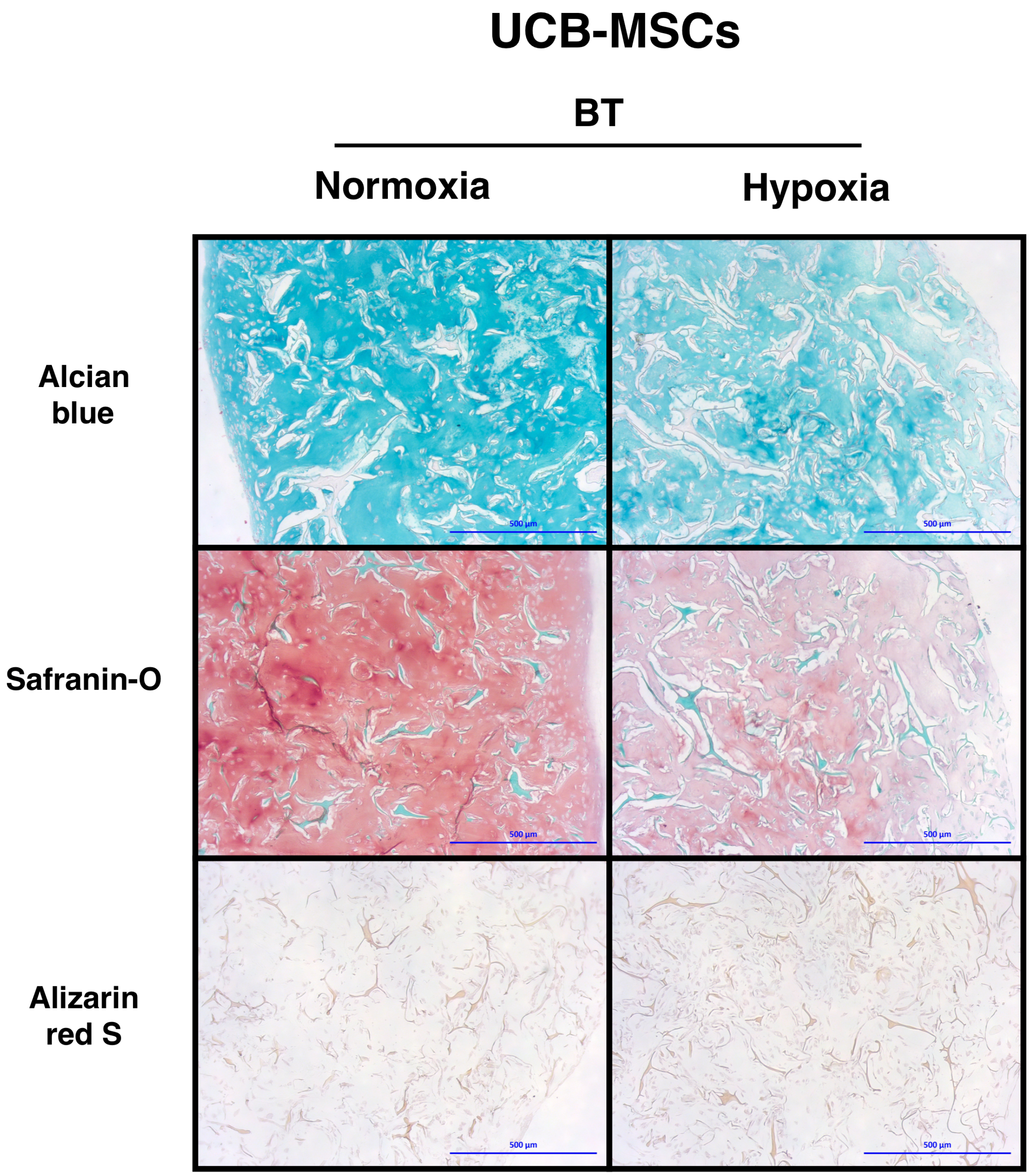

Hypoxia influence on the nature of the matrix newly synthesized by UCB-MSCs under BT treatment.

UCB-MSCs were cultured in type I/III collagen scaffolds at 21% or 3-5% O_2 during 28 days in ICM supplemented with BMP-2 (50 ng/ml) and TGF-ß1 (10 ng/ml). The qualitative nature of the ECM was evaluated by alcian blue (sulfated glycosaminoglycans), O-safranin (sulfated proteoglycans) and alizarine red S stainings (extracellular matrix calcification). A representative example from three samples for UCB-MSCs are shown (magnification: ×10, scale bar: 500 µm).

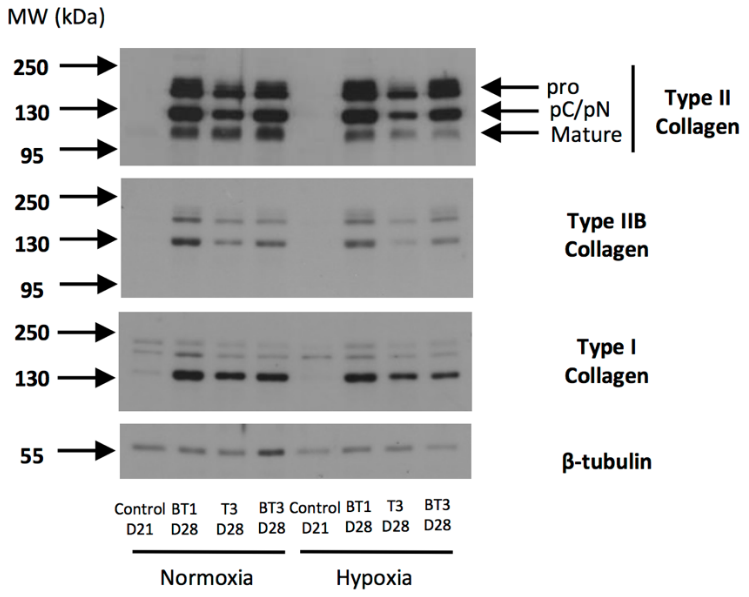

Figure. Comparison between oxygen tension and TGF-β1 or TGF-β3 in addition to BMP-2 on protein expression of equine umbilical cord blood-derived mesenchymal stem cells (eUCB-MSCs) differentiated into chondrocytes. eUCB-MSCs at passage 4 were cultured in type I/III collagen sponges for 21 (D21) and 28 days (D28) in normoxia or in hypoxia, in the absence (control), or in the presence of 50 ng/ml of BMP-2 and 10 ng/ml of TGF-β1 (BT1) or 10 ng/ml of TGF-β3 (T3) or BMP-2 and TGF-β3 (BT3). Protein extracts were analyzed in western blots for type II, type IIB and type I *versus* β-tubulin. Representative blots are shown of two independent experiments. Type II collagen shows different levels of maturation forms such as type II procollagen (pro), with only C- or N-terminal propeptides (pC/pN) and the doubly cleaved form (mature form). MW: Molecular Weight, kDa: kilodaltons.
